# Different Patterns of mRNA Nuclear Retention during Meiotic Prophase in Larch Microsporocytes

**DOI:** 10.3390/ijms22168501

**Published:** 2021-08-07

**Authors:** Karolina Majewska, Patrycja Wróblewska-Ankiewicz, Magda Rudzka, Malwina Hyjek-Składanowska, Marcin Gołębiewski, Dariusz Jan Smoliński, Agnieszka Kołowerzo-Lubnau

**Affiliations:** 1 Department of Cellular and Molecular Biology, Nicolaus Copernicus University, Lwowska 1, 87-100 Torun, Poland; k.majewska@doktorant.umk.pl (K.M.); pw@doktorant.umk.pl (P.W.-A.); madzialenka12@interia.pl (M.R.); mhyjek@iimcb.gov.pl (M.H.-S.); 2 Centre For Modern Interdisciplinary Technologies, Nicolaus Copernicus University, Wilenska 4, 87-100 Torun, Poland; mgoleb@umk.pl; 3 Department of Plant Physiology and Biotechnology, Nicolaus Copernicus University, Lwowska 1, 87-100 Torun, Poland

**Keywords:** mRNA nuclear retention, mRNP, Cajal bodies, retained introns, transcriptional noise, *Larix decidua* Mill.

## Abstract

Recent studies show a crucial role of post-transcriptional processes in the regulation of gene expression. Our research has shown that mRNA retention in the nucleus plays a significant role in such regulation. We studied larch microsporocytes during meiotic prophase, characterized by pulsatile transcriptional activity. After each pulse, the transcriptional activity is silenced, but the transcripts synthesized at this time are not exported immediately to the cytoplasm but are retained in the cell nucleus and especially in Cajal bodies, where non-fully-spliced transcripts with retained introns are accumulated. Analysis of the transcriptome of these cells and detailed analysis of the nuclear retention and transport dynamics of several mRNAs revealed two main patterns of nuclear accumulation and transport. The majority of studied transcripts followed the first one, consisting of a more extended retention period and slow release to the cytoplasm. We have shown this in detail for the pre-mRNA and mRNA encoding RNA pol II subunit 10. In this pre-mRNA, a second (retained) intron is posttranscriptionally spliced at a precisely defined time. Fully mature mRNA is then released into the cytoplasm, where the RNA pol II complexes are produced. These proteins are necessary for transcription in the next pulse to occur.mRNAs encoding translation factors and SERRATE followed the second pattern, in which the retention period was shorter and transcripts were rapidly transferred to the cytoplasm. The presence of such a mechanism in various cell types from a diverse range of organisms suggests that it is an evolutionarily conserved mechanism of gene regulation.

## 1. Introduction

Transcription in eukaryotic cells occurs in a stochastic [1,2,3] and pulse manner [4,5,6], which may generate transcriptional noise. Such transcriptional noise can be a phenomenon that concerns all expressed transcripts or can be limited only to some specific transcripts. In some cases, this phenomenon is beneficial for the cells, but usually, it is undesirable and must be suppressed [7]. It was examined that gene-specific transcriptional noise is responsible for the phenotypic variability of isogenic cell populations living in homogeneous environments. In this situation, the variance of expression is a potential target of adaptation [8]. Positive aspects of this process also occur in clonal populations of bacteria that can diversify cell fates. Transcriptional noise can also be enhanced to achieve competence in *Bacillus subtilis* [9].

In contrast, transcriptional noise, which is caused by pulsed transcription, must constantly be buffering to allow the proper functioning of the cells [7].It is diminished or cancelled out at the post-transcriptional level. The transcriptional burst can be regulated by elements such as changes in chromatin condensation, promoter architecture, or enhancer-promoter interactions [7].

Periods in which a burst of transcriptional activity occurs are separated by phases with no or low transcription [6]. The result is an average constant level of synthesis of specific proteins based on the mRNA pool constantly available in the cytoplasm. One of the main processes that influences the reduction of transcriptional noise is the process of pre-mRNAs and mRNAs retention in the nucleus. Due to this process, there is no simultaneous export of the whole pool of synthetized mRNAs to the cytoplasm. This prevents transcriptional chaos and significant fluctuations in the level of proteins in the cytoplasm. It was examined that post-transcriptional and translational auto-regulatory motifs are more effective at noise silencing than common negative-feedback [10]. The role of mRNAs retention in the cell nucleus in the process of post-transcriptional reduction of transcriptional noise was discovered in the last decade and is now extensively studied. It appears to be common in all eukaryotes, both unicellular, such as yeasts [11] and multicellular plants [12,13] as well as animals [14].

Recent studies suggested another role of nuclear mRNAs retention, namely strict temporal control of specific mRNAs export to cytoplasm [11,12,13,14]. Furthermore, regulation of gene expression by nuclear mRNA retention occurs in mammalian metabolic tissues both under stress [15,16] and normal physiological conditions [14]. It was also found in generative cells that require the expression of specific proteins at a time strictly defined by their developmental stage [11,12,13]. Retained transcripts are localized in the nucleoplasm, nuclear basket, and nuclear domains such as nuclear speckles, paraspeckles, Cajal bodies, and nuclear foci (“dots”) [13,17,18,19,20]. mRNA retention may be due to an unspliced intron, a poly(A) tail with an altered length, cis-elements, or convert adenosine to inosine [13,17,18,19,20]. Transcripts accumulated in nuclear foci in *S. cerevisiae* have a hippo- or hyperacetylated poly(A) tail [19]. Inhibited splicing via the attachment of spliceostatin A to the SF3b sub-complex in U2 snRNP causes the retention of transcripts with unspliced intron and its accumulation in the nuclear domains [20]. Early spliceosome elements such as U1 snRNP and U2 snRNP are usually attached to the unspliced intron, resulting in the accumulation of these transcripts in nuclear speckles [18,21]. mRNAs remain in the nucleus for a long time, and their export to the cytoplasm is slow. This slow export may be caused by a nuclear zip code, an RNA segment in ORF in mRNA [22]. Removal of retention inducers results in rapid nuclear RNA export and translation [19,22]. The increased speed of nuclear export may be caused by modifications of mRNA, e.g., N6-methyladenosine (m6A) and N5-methylcytosine [18,23].In mammalian cells that show a high level of metabolic activity (liver, gut, and beta cells), transcripts are stored for several hours before being transported to the cytoplasm [13]. In mouse liver cells, the sequence coding for mCAT2 protein is “enclosed” in a long non-coding RNA (CTN-RNA) [16]. Stress causes cleavage in the 3′ UTR region and export of mRNA to the cytoplasm and, consequently, protein synthesis. As a result, the cells are able to respond very quickly to stressful conditions. Similarly, in plants under stress conditions such as hypoxia shock, mRNAs are temporarily retained in the nucleus and are released into the cytoplasm only when hypoxia subsides, when cells return to normal metabolism. Then, there is a rapid transport of mRNA to the cytoplasm and protein synthesis [24]. The molecular basis of this phenomenon has not yet been understood.

In generative cells, the process of nuclear mRNAs retention appears to be associated with periods of genome silencing. Such a phenomenon was observed during meiosis in yeast [11], spermatogenesis in animals [14], and during meiosis and the development of microsporocytes in plants [12,13]. In all these cases the mechanism involves intron retention. In pre-mRNAs usually only one or two introns are left and then post-transcriptionally spliced when a cell needs mature mRNAs.

Previously, we demonstrated that during meiosis in larch microsporocytes, regulation of gene expression by nuclear mRNA retention occurred. During the first meiotic prophase we observed 5 cycles of transcriptional activity and 4 periods of transcriptional silencing related to chromatin contraction during the diplotene stage (lasting about 5 months) [25]. We observed accumulation of mRNAs in the nucleoplasm and Cajal bodies (Cbs) in each of these cycles [24]. The site of retention of pre-mRNAs with conserved intron sequences are CBs, and the introns are removed before mRNAs are exported to the cytoplasm. 

In this study, we analysed the duration of a single cycle synthesis and flow of RNA poly (A) in microsporocytes. We also performed an analysis of the transcriptome of these cells. Based on the analysis, mRNAs related to various metabolic processes of these highly active generative cells were selected. We present different expression profiles for these mRNAs: patterns of nuclear retention and transport to the cytoplasm. We also try to explain the physiological sense of RNA pol II subunit 10 transcript retention and delayed expression during the subsequent stages of meiotic prophase.

The innovation of this study is related to the involvement of the CBs in the regulation of gene expression viamRNA retention. This phenomenon has not yet been described in any other model. Our research has also shown the existence of different patterns of mRNA nuclear retention and export. The results of conducted analyses can help better understand the processes involved in the maturation of germ cells in plants and post-transcriptional regulation of mRNA expression in eukaryotic cells.

## 2. Results

**Figure 1:**
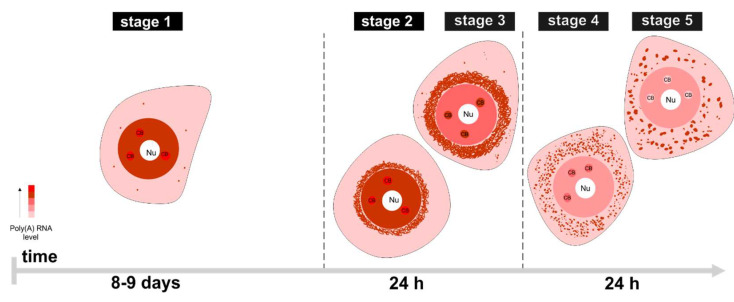
The stages of poly(A) RNA cycle. Poly(A) RNA is localized almost exclusively in nucleus (both in nucleoplasm and Cajal bodies (CBs)) during the first stage in which transcription occurs. At the second and third stage poly(A) RNA is transferred to cytoplasm; during the former, poly(A) RNA is still present in nucleus in large quantities, while during the latter stage, the majority is already exported. The fourth and fifth stage are characterized by low levels of poly(A) RNA in nucleoplasm and high levels in cytoplasm, and in the fifth one there is no polyadenylated RNA in CBs. CB—Cajal body, Nu—nucleolus.

Our previous studies have shown that mRNA synthesis in larch microsporocytes occurs in a pulsatile manner. Certain mRNAs remain in the nucleus for a long time after synthesis. They are then successively transported to the cytoplasm where they are translated [13]. In previous studies, we have analyzed a single cycle of cellular synthesis and turnover of poly(A) RNAs, which allows us to distinguish successive stages characterized by changes in the level and distribution of poly(A) RNA [13]. In the present study, we focused on determining the developmental synchronicity of microsporocyte development during meiosis and on the exact duration of each stage of the cycle. We observed that larch microsporocytes were developmentally synchronous both within a single flower bud (93.1%) (Table S1, Figure S1) and buds from a single branch (92.9%) (Table S2, Figure S2). Therefore, to determine how long each stage lasts, individual flower buds were harvested every day, protoplasts were isolated, and poly(A) RNA fluorescent in situ hybridization (FISH) was performed to identify the specific stage. A single cycle lasted in the range of10–11 days, of which the longest stage, lasting 8–9 days, was the first one, in which poly(A) RNAs were synthesized and retained in the cell nucleus (Figure 1).

The export of poly(A) RNA to the cytoplasm starts at the beginning of the second stage and then proceeds very quickly. The next steps last only a few hours each. This indicates that when expression of mRNA starts to be needed, the next steps take place very quickly [13].

**Figure 2:**
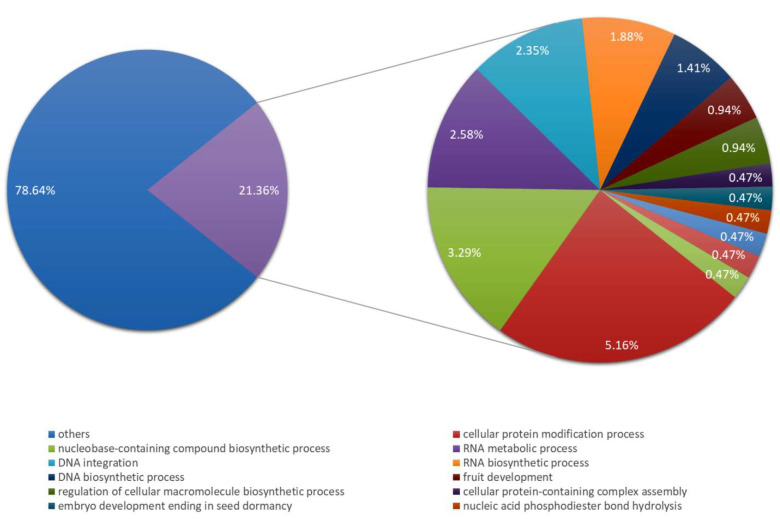
Breakdown of transcripts with GO terms from sixth level of ‘Biological Process’ domain. In the circle on the right, the most common transcripts from the category of Biological Process.

Our research so far has mainly shown the fate of the general pool of polyadenylated transcripts during a single cycle of mRNA synthesis and turnover. Here, we made an attempt to check how such a cycle proceeds for individual mRNAs. To get information on which mRNAs were synthesized in a single cycle, we sequenced, assembled, and analyzed the transcriptome of larch microsporocytes. We obtained 220,400 transcript sequences over 150 nt in length. To assign putative functions, potential homologs were identified by searching NCBI nr or ntdatabases using BLASTX (for potential mRNA sequences) or BLASTN (for other sequences), respectively. GO-terms assignment was performed for protein coding sequences, for which 1024 transcripts with the highest expression over the studied period were selected. 214,112 GO terms were assigned to 426 sequences (Supplemental Dataset 1). In the “biological process” domain at the sixth level (chosen to show detailed categories) the majority of transcripts were assigned terms from categories related to nucleic acids metabolism (Figure 2).

We chose mRNAs involved in the most important biological processes (identified based on GO categories and enumerated below) to see how they were expressed relative to the total poly(A) RNA pool. We tracked thirteen mRNAs, encoding the following proteins: (1) RNA metabolism: SNRP27—homolog of small nuclear ribonucleoprotein 27, RPB10—polymerase II RNA subunit, PABP4—poly(A) tail 4 binding protein, SERRATE—involved in alternative splicing and involved in the microRNA biogenesis pathway, (2) translation related: RS6—S6 small ribosomal subunit protein, eF1a—translation elongation factor 1a, eIF5b—translation initiation factor 5b, (3) associated with the cytoskeleton: ACT—actin, CLATH—clathrin, (4) organelle organization related: PER40—peroxidase 40, SDH—succinate dehydrogenase, PEP—plastid RNA polymerase, (5) stimulus response related: DNJ—protein with DNaJ domain. 

Based on the time spent in the nucleus and the initial moment of export, we identified two main patterns of mRNA synthesis and export. 

**Figure 3:**
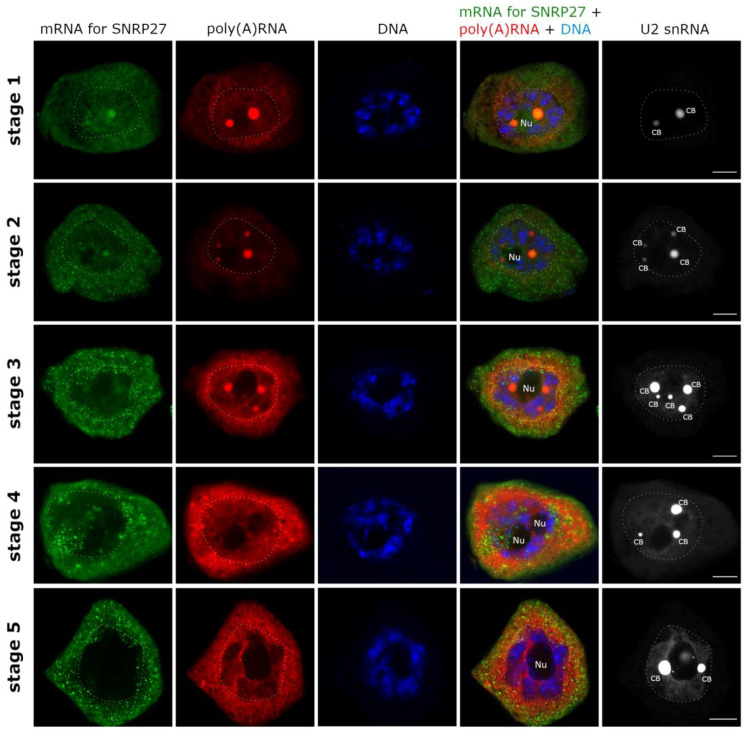
The first pattern of mRNA synthesis and export. Distribution of SNRP27 mRNA during poly(A) RNA cycle. SNRP27 mRNA (green) remains in the nucleus up to the 4th stage of poly(A) cycle. The exportstarts in the first stage and SNRP27 mRNA is present in cytoplasm until the end of the cycle. The transcript is localized to Cajal bodies (CBs) from the first to third stage of the cycle. SNRP27 mRNA (green), Poly(A) RNA (red), DNA (blue), U2 snRNA (white). The border between the nucleus and the cytoplasm is marked with a white dashed line. Bar—10 μm.

The first of the patterns was characterized by a long retention time, until the third/fourth stage of the cycle and export beginning in the first/second stage. This category included mRNAs encoding: SNRP27, PER 40, ACT, DNJ, PEP, PABP4, RPB10 (Figure 3 and Figure S3).

**Figure 4:**
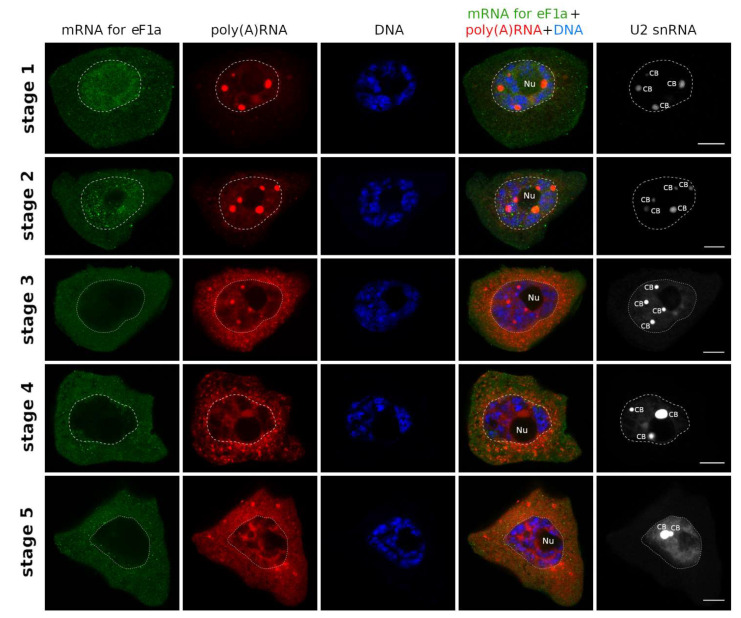
The second pattern of mRNA synthesis and export. Distribution of eF1a mRNA during poly(A) RNA cycle.eF1a mRNA (green) is retained in nucleus until the 2nd stage of poly(A) cycle and is rapidly exported to cytoplasm at the beginning of stage 3. The mRNA is present in cytoplasm until the end of the cycle. Bar—10 μm.

The second pattern was characterized by a shorter retention period (until second/third stage) and later beginning of export (second stage). This expression profile was characteristic of mRNAs coding for translation factors: RS6, eF1a, eIF5b, Serrate (Figure 4 and Figure 6, Figure S4). In both patterns the cytoplasmic concentration of a given mRNA increased over time until the very end of the cycle.

In addition to these two dominant patterns, we also observed others deviating from them, which suggested the possibility of precise adaptation of the system to the demand for a specific protein at the right time (Figures S5 and S6).

**Figure 5:**
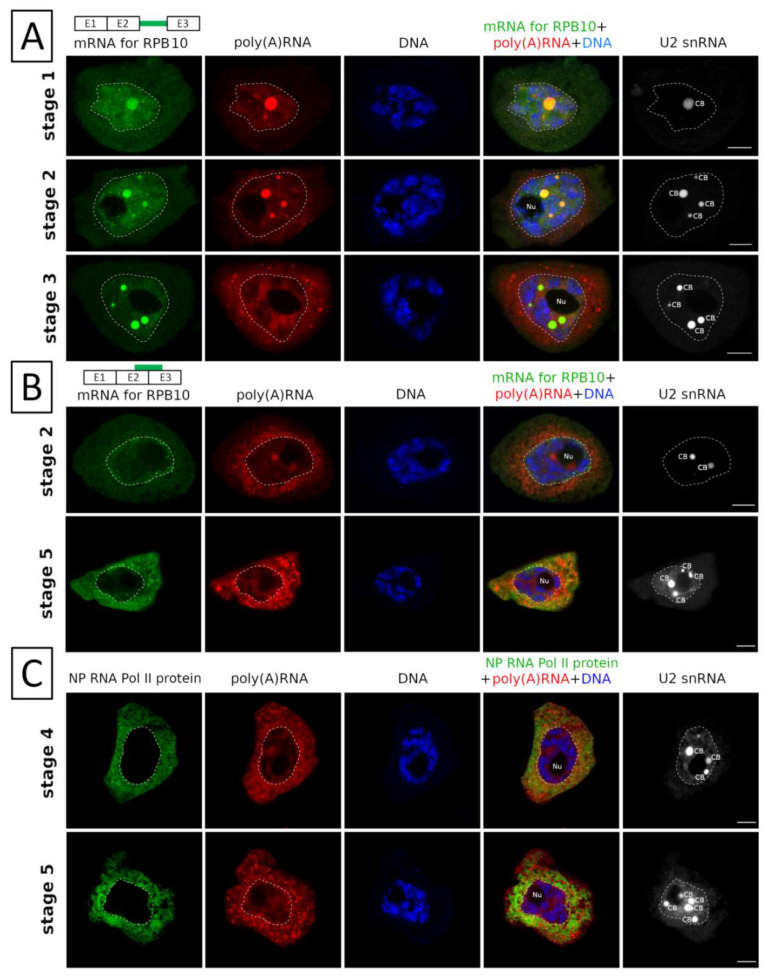
Detailed analysis of the mRNA encoding the RNA polymerase II and non-phosphorylated (NP) RNA pol II protein. (**A**) Distribution of the pre-mRNA encoding the RNA polymerase II with the second retained intron against the background of the pool of poly(A) RNA. U2 snRNA staining was used to show Cajal bodies (CB). Labelling of the second intron sequence present in the mRNA (green) showed that this sequence is accumulated in the nucleoplasm and CBs from the first to the third stage of poly(A) RNA cycle. The symbols E1, E2, E3 represent the first, second and third exons of the transcript. The green line marks the second (retention) intron sequence to detect which complementary probe was used. (**B**) After removal of the retained intron, the mRNAs are no longer accumulated in CBs. Mature RNA Pol II-encoding subunit 10 mRNA is absent from CBs during all stages. During 1st and 2nd stage it is present in the nucleoplasm, concentrates at nuclear membrane in stage 2, while during stages 4 and 5 it is present in cytoplasm. The green line marks the sequence in the second (E2) and third (E3) exons. The complementary probe to that sequence hybridizes, detecting the transcript after splicing the second intron. (**C**) Retained mRNAs are exported to the cytoplasm and translated. New non-phosphorylated RNA pol II proteins are formed in the cytoplasm in large amounts, from the mRNA pool that was processed in the cycle. Nu-nucleolus, CB—Cajal body. Bar—10 µm.

Our previous studies have shown that, in the case of mRNAs encoding Sm proteins, there is nuclear retention of not fully spliced transcripts with one or more introns unspliced. We performed a detailed analysis of the mRNA encoding the RNA polymerase II subunit 10, the key protein for the expression of protein-encoding genes, and checked if it was subject to the same type of regulation. As mentioned before, this mRNA belongs to the group displaying the first synthesis and export pattern. The transcript with an unspliced second intron underwent nuclear retention (Figure 5A). 

Unspliced pre-mRNAs are stored in nucleoplasm and, in particular, in Cajal bodies for a long period of time. Fully mature transcripts (after splicing of the second intron) appeared in the nucleus in the second stage of the cycle (Figure 5B). Concentration of the transcript at the periphery of the nucleus was visible, which suggested preparation for export to the cytoplasm. In subsequent stages, the amount of RPB10 transcript in the cytoplasm increased until the fifth stage, when the signal was the strongest. 

The appearance of a large amount of the transcript in the cytoplasm correlated with the appearance of a large pool of non-phosphorylated form of the protein in this compartment (Figure 5C). These data suggested that this was the point at which the individual subunits were translated and the ready-to-use enzymes were assembled.

**Figure 6:**
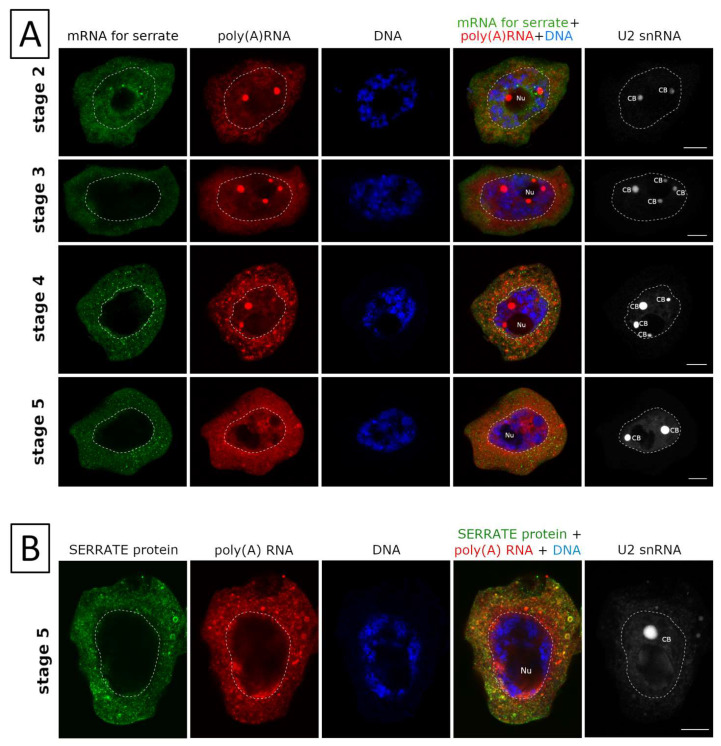
Analysis of SERRATE mRNA and SERRATE protein during poly(A) RNA cycle.(A) SERRATE mRNA (green) is retained in the nucleus until the second stage of the poly(A) cycle and is rapidly exported to the cytoplasm at the beginning of stage 3. The mRNA is present in the cytoplasm until the end of the cycle. (B)Retained mRNAs are exported to the cytoplasm and translated at the end of the cycle. SERRATE proteins are formed in the cytoplasm in large amounts, from the mRNA pool that was processed in the cycle. Nu—nucleolus, CB—Cajal body. Bar—10µm.

A similar pattern was observed with the expression of a SERRATE protein. mRNA encoding this protein represents the second pattern of synthesis and export. Mass export of mRNA encoding the SERRATE protein to the cytoplasm in the 3rd and 4th stages of the cycle (Figure 6A) preceded the appearance of the SERRATE protein in the cytoplasm at the end of the cycle (Figure 6B).

## 3. Discussion

### 3.1. mRNAs Differ in Nuclear Retention Times and Rates of Export to Cytoplasm

Larch microsporocytes are characterized by pulsed transcription [25]. Each such transcriptional burst generates a pool of polyadenylated transcripts, which are initially retained in the nucleus and then gradually released into cytoplasm [13]. Such nuclear mRNA retention is a kind of buffer for transcripts that can be used at a specific point in time. This form of gene expression regulation allows the occurrence of gene expression noise to be prevented [14,26,27,28]. 

The cycles of cellular synthesis and turnover of poly(A) RNAs observed in microsporocytes reflect the discharge of a “traffic jam” generated by a transcriptional burst. In this work, we examined the process in detail for a group of thirteen transcripts encoding proteins involved in various biological processes present in microsporocytes transcriptome. Among them there are transcriptional, splicing and translational factors, factors related to vesicular transport and cytoskeleton as well as organelle metabolism. We showed that different mRNAs differ in their nuclear retention time as well as the time and rate of export to cytoplasm. Some of them are retained in the nucleus for almost the entire cycle and slowly ‘leak’ into the cytoplasm. Others, in turn, stay in the nucleus for a much shorter time and their export to the cytoplasm is delayed but rapid. Such variation indicates the precise regulation of appropriate proteins formation at a specific point in time. This is particularly important in the case of developing or specialized cells that often have completely inhibited transcriptional activity and use a buffer of previously synthesized transcripts.A phenomenon similar to that observed in larch was described during the development of generative cells in both plants and animals. In *Marsileavestita* microspores, intron-containing transcripts (IRTs) are accumulated in the nucleus and are not exported to the cytoplasm until the introns are removed [12]. In this way microspores regulate the removal of retained introns at specific times during development as a means of promoting the translation of proteins needed at a particular stage. A similar mechanism was also observed in mouse meiotic spermatocytes and post-meiotic spermatids [29]. In these meiotic cells, IRT-containing transcripts encode critical proteins for the spermatogenesis process. These pre-mRNAs are synthesized in spermatocytes and are preserved until the late stages of spermatogenesis.

Such transcriptional noise can be a phenomenon that concerns all expressed transcripts or can only be limited to specific transcripts. In some cases, this phenomenon is beneficial for the cells, but usually, it is undesirable and must be suppressed [7]. It was examined that gene-specific transcriptional noise is responsible for the phenotypic variability of isogenic cell populations living in homogeneous environments. In this situation, the variance of expression is a potential target of adaptation [8]. Positive aspects of this process also occur in clonal populations of bacteria that can diversify cell fates. Transcriptional noise can also be enhanced to achieve competence in *Bacillus subtilis* [9].

In contrast, transcriptional noise, which is caused by pulsed transcription, must constantly be buffering to allow the proper functioning of the cells [7]. It is diminished or cancelled out at the post-transcriptional level. The transcriptional burst can be regulated by elements such as changes in chromatin condensation, promoter architecture, or enhancer-promoter interactions [7].

Studies in mouse tissues (such as beta cells, liver, orintestinal tissue) in which the transcription occurs in a pulsating manner showed a wide range of polyadenylated, spliced, protein-coding mRNAs that are nuclearly retained for the majority of their lifetime [13]. This study suggested that nuclear retention of mRNA could buffer cytoplasmic transcript levels from noise that arises from transcriptional bursts.

### 3.2. Two Patterns of Nuclear mRNA Retention in Larch and the Importance of This Process at the Time of the Prophase of the 1st Meiotic Division

In larch microsporocytes a number of transcripts that are products of housekeeping genes, such as mRNAs for SNRNP27, PABP4, ACT, or RPB10, are subject to regulation through nuclear retention. Detailed analysis of the mRNA encoding the 10th subunit of RNA polymerase II showed that this mRNA is retained in a non-fully spliced form with a single intron unspliced. This form is stored in the nucleus almost until the end of the cycle. Appearance of its fully mature form in the nucleus in the second stage of the cycle initiates its export to the cytoplasm, which continues until the end of the cycle. The last stages of the cycle, when the cytoplasmic pool of mRNAs is largest, are the moment when a new pool of the protein appears in the cytoplasm. At this time, when the pool of cytoplasmic mRNA coding for RPB10 is the highest, RNA pol II subunits are massively translated in the cytoplasm. It happens when there is demand for fully functional polymerase to be used in a new transcriptional burst at the beginning of a new cycle. The next transcriptional burst cannot be performed without a supply of RNA polymerase II, while the level of polymerase depends on the retention of mRNA encoding its subunit (or subunits) and its rapid transport to the cytoplasm at the right time. The mechanism described above shows how demand for a functional multi-subunit complex at a given time might be fulfilled by retention of mRNA coding for a single subunit. It is possible that other subunits are regulated in a similar manner. 

There is one more important element that may regulate the global nuclear retention of mRNAs. The mRNAs encoding translation factors RS6, eF1a, eIF5b were classified into the second pattern of mRNAs synthesis and export. They stayed in the nucleus for a relatively short time, until the second‒third stage, but what is most interesting is that their export to the cytoplasm began only in the second stage and was rapid. A delay in the synthesis of translation factors will affect the translation of mRNAs, which will further affect the availability of other proteins. The facts described above suggest that translation also occurs in bursts temporally correlated with transcription bursts. 

In our previous studies [13] we observed retention of mRNAs encoding Sm proteins involved in splicing. These mRNAs underwent nuclear retention as a result of preservation of the intron sequence and, like the mRNA encoding RPB10, were present in immature form in the nucleus almost throughout the cycle. Retention of these coding mRNAs contributed to a deficiency of splicing factors, which could have affected the nuclear retention of other mRNAs. This phenomenon was confirmed in mice during granulopoiesis [30]. In order to check whether changes in the splicing machinery could cause intron retention, the expression of mRNAs and the level of proteins contained in small nuclear ribonucleoproteins, which build spliceosomes, were examined. The majority of the U1 and U2 subunits were downregulated during granulopoiesis, whereas the other subunits were mainly stable. The level of Sf3b1, essential for intron definition, decreased seven-fold [31]. This indicated that IR might arise from the downregulation of spliceosomal components responsible for recognizing intron and exon boundaries, especially at the branch point and the 5′ extremity of the intron.Nuclear retention of mRNAs seems to be a mechanism that regulates protein synthesis so that specific ones can appear at the right moment in the sufficient quantity required by the metabolic needs of rapidly developing cells (during diplotene these cells increase their volume up to sixtimes [25]).By creating a buffer in the form of mRNAs accumulated in the nucleus, cells can produce proteins without resuming transcriptional activity. It also seems that the retention process itself is regulated by many feedback loops that affect the availability of appropriate elements of this system. A detailed explanation of all mechanisms of this process remains open-ended and is linked to further extensive research.

## 4. Materials and Methods

### 4.1. Plant Materials and Isolation of Meiotic Protoplasts 

Anthers of European larch (*Larix decidua* Mill.) was collected from the same tree (Toruń, Poland, coordinates 53.021155 N, 18.570384 E) in the diplotene stage of prophase of larch microsporocytes from December to January at weekly intervals. They were fixed in 4% paraformaldehyde solution in phosphate-buffered saline (PBS) buffer pH 7.2 for 12 h and squashed to obtain free meiocytes. Meiotic protoplasts were isolated from these cells according to the method of Kołowerzo et al. [32]. Isolated protoplasts were next used for fluorescent in situ hybridization (FISH). 

### 4.2. FISH in Multiplex Reactions 

Prior to the assay, the cells were treated with 0.1% Triton X-100 in PBS for 10 min. For the hybridization, the probes were resuspended in hybridization buffer (30% *v/v* formamide, 4× SSC, 5× Denhardt’s buffer, 1 mM EDTA, 50 mM phosphate buffer) at a concentration of 50 pmol/mL. Hybridization was performed overnight at 27 °C. The sequences of all oligo probes used in this work are summarized in Table 1. The antisense DNA oligonucleotides (Genomed, Warsaw, Poland) were dissolved in the hybridization buffer at a concentration of 10 pmol/mL.Digoxygenine (DIG) probes were detected after hybridization using rabbit anti-DIG antibody (Thermo Fisher Scientific, Waltham, MA, USA) diluted in 0.01% acetylated BSA in PBS (1:100) in a humidified chamber kept at 11 °C overnight and secondary anti-rabbit antibody labelled with Alexa 488 (Invitrogen) diluted in 0.01% acetylated BSA in PBS (1:100) in a humidified chamber kept at 33 °C for 1.5 h. After labelling, the slides were stained for DNA detection with Hoestch (1:1000) and mounted in ProLong Gold Antifade reagent (Life Technologies, Carlsbad, CA, USA).

### 4.3. Immunodetection of Non-Phosphorylated RNA Pol II and SERRATE Proteins

Prior to the assay, the cells were treated with 0.1% Triton X-100 in PBS for 25 min to induce cell membrane permeabilization. Nonspecific antigens were blocked with PBS buffer containing 2% acetylated BSA for 15 min. Non-phosphorylated CTD domain of RNA pol II and SERRATE proteins was detected by incubating respectively with rat anti-RNA pol II (Chromotek, Planegg-Martinsried, Germany; 1:100) and rabbit antibodies (Agrisera, Vännäs, Sweden; 1:250) in 0.01% acBSA in PBS, pH 7.2 (overnight incubations). After rinsing with PBS, the samples were incubated for 1 h at 37°C with the following secondary antibodies: anti-rat Alexa 488 (Thermo Fisher Scientific, Waltham, MA, USA) or anti-rabbit Alexa 488 (Thermo Fisher Scientific, Waltham, MA, USA) 1:500 in 0.01% acBSA in PBS, pH 7.2. 

### 4.4. Design of Multi-Labelling Immunofluorescence-FISH Reactions

Doublelabellingof immunofluorescence-FISH reactions (non-phosphorylated RNA pol II proteins + poly(A) RNA + U2 snRNA; SERRATE proteins + poly(A) RNA + U2 snRNA) wasperformed as described above. In the doublelabelling of immunofluorescence-FISH reactions, the immunocytochemical methods always preceded the in situ hybridization methods because when in situ hybridization was applied first, the subsequent levels of immunofluorescence signals were very weak.

### 4.5. Confocal Microscopy

The images were captured with a Leica TCS SP8 or Olympus FV3000 confocal microscope. The optimized pinhole, long exposure (400Hz), and 63X (numerical aperture 1.4) Plan Apochromat DIC H oil immersion lens were used. The images were collected sequentially in the blue (Hoechst 33342), green (Alexa 488), red (Cy3), and far red (Alexa 633, Cy5) channels. To minimize bleed-through between the channels, we employed low laser power (1.5% of maximum power) and sequential collection. For all antigens and developmental stages, the obtained data were corrected for background autofluorescence, as determined by negative control signal intensities. ImageJ [33] was used for image processing and analysis. Each reaction step was performed using consistent temperatures, incubation times and concentrations of probes and antibodies. 

### 4.6. Protoplasts of a Single Bud and Single Branch, Analysis of Synchronicity and Duration of Stages 

To investigate the developmental synchronicity of larch microsporocytes within one flower bud, protoplasts were prepared in accordance with the procedure described earlier. In this case, only one bud was used. Cells were resuspended in 80 μL of PBS buffer. The entire volume of the suspension was placed on two slides (40 μL for each slide). To investigate the developmental synchronicity of larch microsporocytes within one branch; protoplasts were prepared in accordance with the procedure described earlier. In this case, the cells were isolated from 10 flower buds coming from one branch. Cells were resuspended in 40 μL of PBS buffer. The entire volume was placed on one slide. In both cases on such prepared protoplasts, double-labelling was performed. The hybridisation reaction was carried out in accordance with the previously described procedure. Directly labelled probes were used: poly(A) RNA probe with Cy3 and U2 snRNA probe with Cy5. Images were collected using the Leica Sp8 confocal microscope. 

The collection of data was carried out by counting the cells at different stages of the poly(A) RNA cycle. To ensure reliable results, multiple counting of one cell was prevented by marking counted ones on a slide map.

### 4.7. Libraries Preparation and Sequencing 

RNA from microsporocytes was isolated with aPicoPure RNA Isolation Kit according to the manufacturer’s protocol. After qualitative (Bioanalyzer 2100; Agilent Technologies, Santa Clara, CA, USA) and quantitative (NanoDrop 2000, Qubit; Thermo Fisher Scientific, Waltham, MA, USA) evaluation, the RNA was used for library preparation. Libraries wereprepared with the use of a Total RNA Seq Library Preparation Kit (Lexogen, Vienna, Austria) according to the manufacturer’s protocol, but with displacement stop primers (DSPs) concentration decreased by half (0.5 µL per reaction), which resulted in longer library fragments. Quantification of the libraries was done viaqPCR with a KAPA Library Quantification Kit for Illumina (KAPA Biosystems, Wilmington, MA, USA). The analyses were performed in accordance with the manufacturer’s protocol. Next the libraries were sequenced on MiSeq (Illumina, San Diego, CA, USA) with MiSeq Reagent Kit v3 (600 cycles, Illumina, San Diego, CA, USA). 

### 4.8. Bioinformatic Analysis

The reads were filtered in terms of quality via PRINSEQ-lite [34]. The reads from all the libraries (we have created libraries for 16 biological repetitions) were pooled and assembled with Trinity [35]. Next the sequence of transcripts wasanalysed with BLASTX (for putative mRNA) and BLASTN (for other RNA) with the use of the NCBI databases. Putative functions and ontologies were found using Blast2GO [36] based on BLAST results. 

## 5. Conclusions

Two patterns of mRNA nuclear retention and export to cytoplasm were observed. The majority of the studied transcripts followed the first one, consisting ofof a long retention period and slow release to the cytoplasm. mRNAs encoding translation factors and SERRATE followed the second pattern, in which the retention period was shorter and transcripts were rapidly transferred to the cytoplasm. The transcript encoding subunit 10 of RNA pol II was regulated according to the first pattern, which might constitute a mechanism ensuring tight transcription control due to negative feedback.

## Figures and Tables

**Table 1:** The sequences of the used probes for the FISH reaction.

Probe	Sequence
total poly(A)—RNA	5′ Cy3 T(T)29
U2 snRNA	5′ Cy5 ATATTAAACTGATAAGAACAGATACTACACTTG 3′
mRNA for eF1a	5′ DIG TGGTGACCTTTGCACCAGTGGGATCCTTCT 3′
mRNA for ACT	5′DIG TCTTCTGGAGCAACTCGAAGCTCATTGTAGA 3′
mRNA for eIF5b	5′DIG TCTTCACACCAAGTTCATCTGCAAGCTCACGAG 3′
mRNA for SDH	5′ DIG ACTTGTTCGTCTCTTCATAGACCTCCCCACCAAT 3′
mRNA for CLATH	5′DIG TGCAAGATTCACAGCAAGCTCTAGATTATTCAGC 3′
mRNA for SNRP27	5′DIG TCGGAATCCCCAATTTCTTCATCATCTC3′
mRNA for RPB10 intron 2	5′Dig AAAAGCAAAGATAAGACGATTGATG3′
mRNA for RPB10 on the border of exon2/exon3	5′Dig CACTCCTCTCTAAAGTGTTGTAATTCAAAA 3′
mRNA for PEP	5′Dig TGTAACCCGTGCATAAGGTCCACTCGAACGTGC 3′
mRNA for DNJ	5′Dig TCTTTAAGGGCATCCTCTCCATACTGGTCA 3′
mRNA for PABP4	5′Dig ACTCCATAAAGCCATAGCCCTCTGACTGTCCA 3′
mRNA for RS6	5′Dig TTCCTTTGCAGAGTCAATGGTGTCACAAGCC 3′
mRNA for PER40	5′Dig TGAATGTTGCACAACGTGCCTTTCCTATTGTA 3′
mRNA for SERRATE	5′Dig GTTCCATAGTAATCCAATCCATGAACTCGCCA 3′

## Data Availability

Data supporting the findings of this work are provided in the main text and the supporting information files. Raw reads generated during this project will be available at request from the corresponding authors. The full dataset for the transcriptomic analysis (larch meiocytes 220 400 sequences over 150 nt in length) are available at https://data.mendeley.com/drafts/r6p47rh7xx/1 (accessed on 21 Jun 2021) [37]. All other data and materials used in this study will be available at request from the corresponding authors.

## Supplementary Materials

The following are available online at https://www.mdpi.com/article/10.3390/ijms22168501/s1.
