# Supplementary material for: Different Patterns of mRNA Nuclear Retention during Meiotic Prophase in Larch Microsporocytes
Source: Int J Mol Sci. 2021 Aug 7;22(16):8501. doi: 10.3390/ijms22168501 (PMC8395157; doi:10.3390/ijms22168501)
Supplement: Supplementary file 1 [file ijms-22-08501-s001.zip › Supplemental Figures Majewska et al 2021.pdf]

Supplemental figures.

Stage	Number of cells	Percentage
1	469	93,1 %
5	35	6,9 %
**Total**	**504**	**100 %**

Table S1. Synchronicity of microsporocytes within a single flower bud.

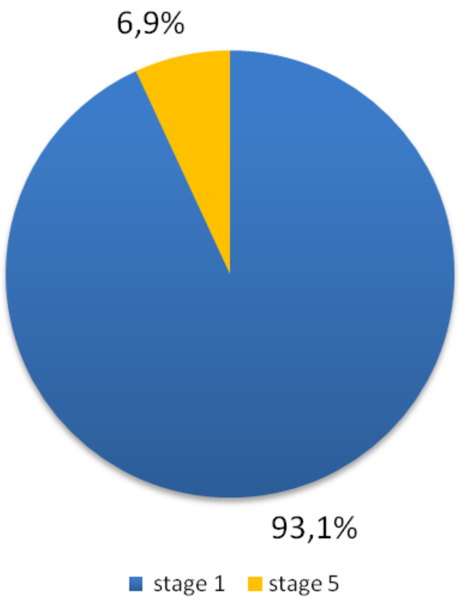

Figure S1. Graph showing percentage of identified stages for microsporocytes within a single flower bud.

Stage	Number of cells	Percentage
1	663	92,9%
2	8	1,1 %
3	21	2,9 %
4	7	1,0 %
5	15	2,1 %
**Total**	**714**	**100%**

Table S2. Synchronicity of microsporocytes within a single branch

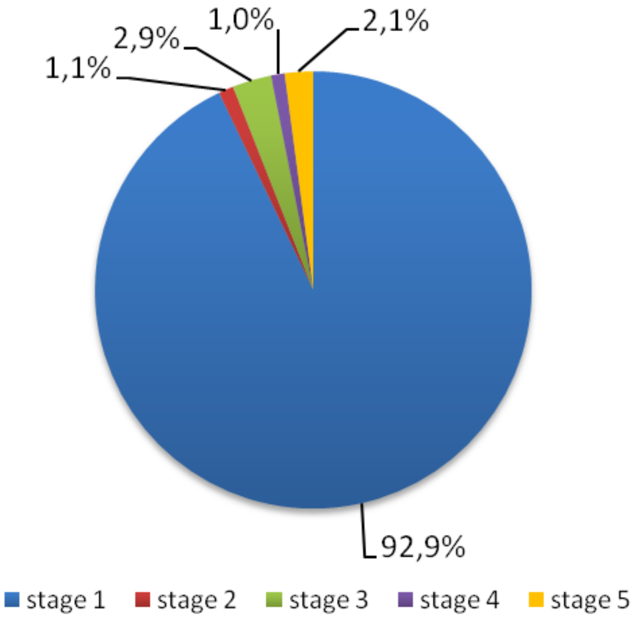

Figure S2. Graph showing percentage of identified stages for microsporocytes within single branch.

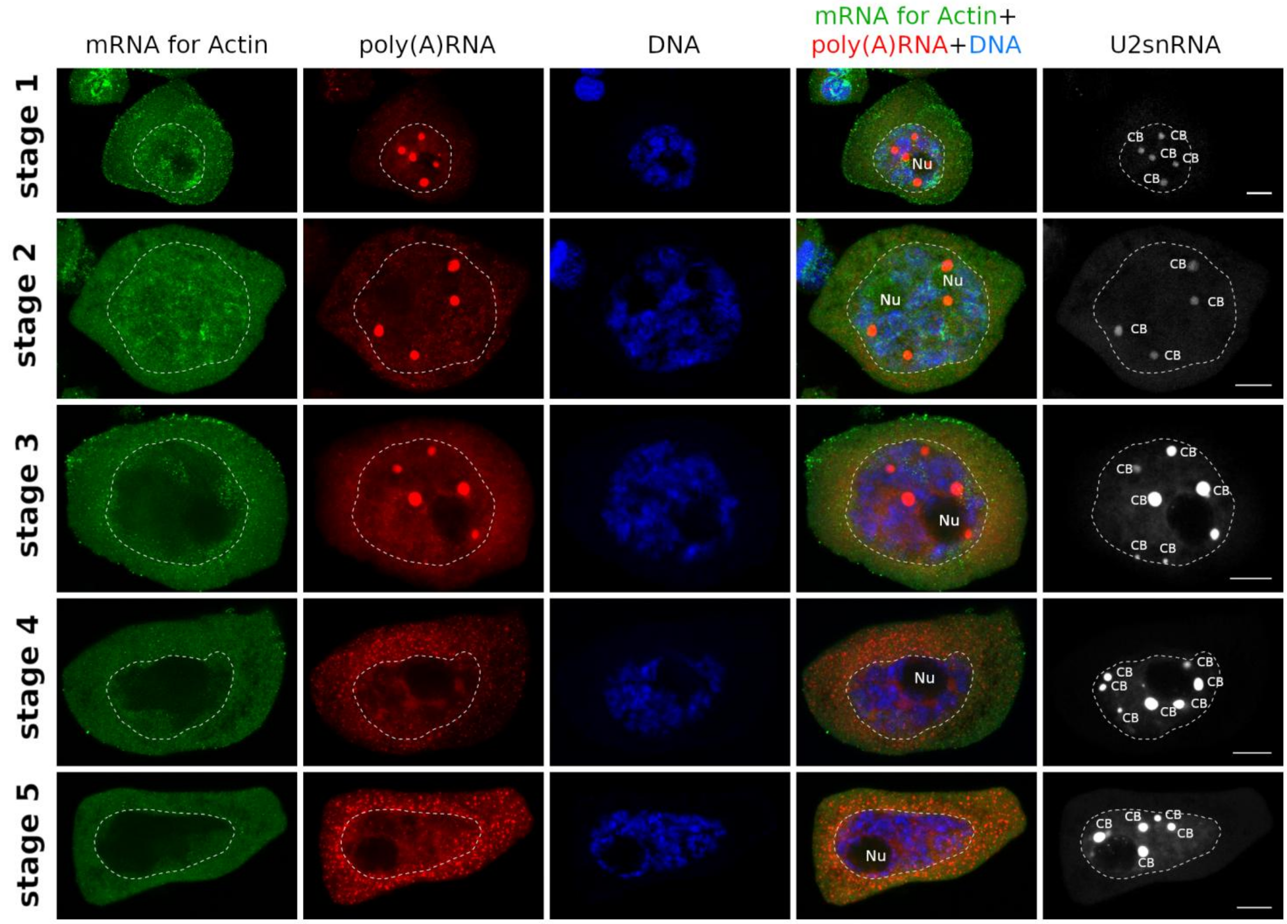

Figure S3. The first pattern of mRNA synthesis and export. Distribution of actin mRNA during poly(A) RNA cycle. Actin mRNA (green) remains in the nucleus for a long time, up to the 4th stage of the cycle, and export starts in the first stage. The mRNA is present in the cytoplasm throughout the whole cycle. Bar – 10 μm.

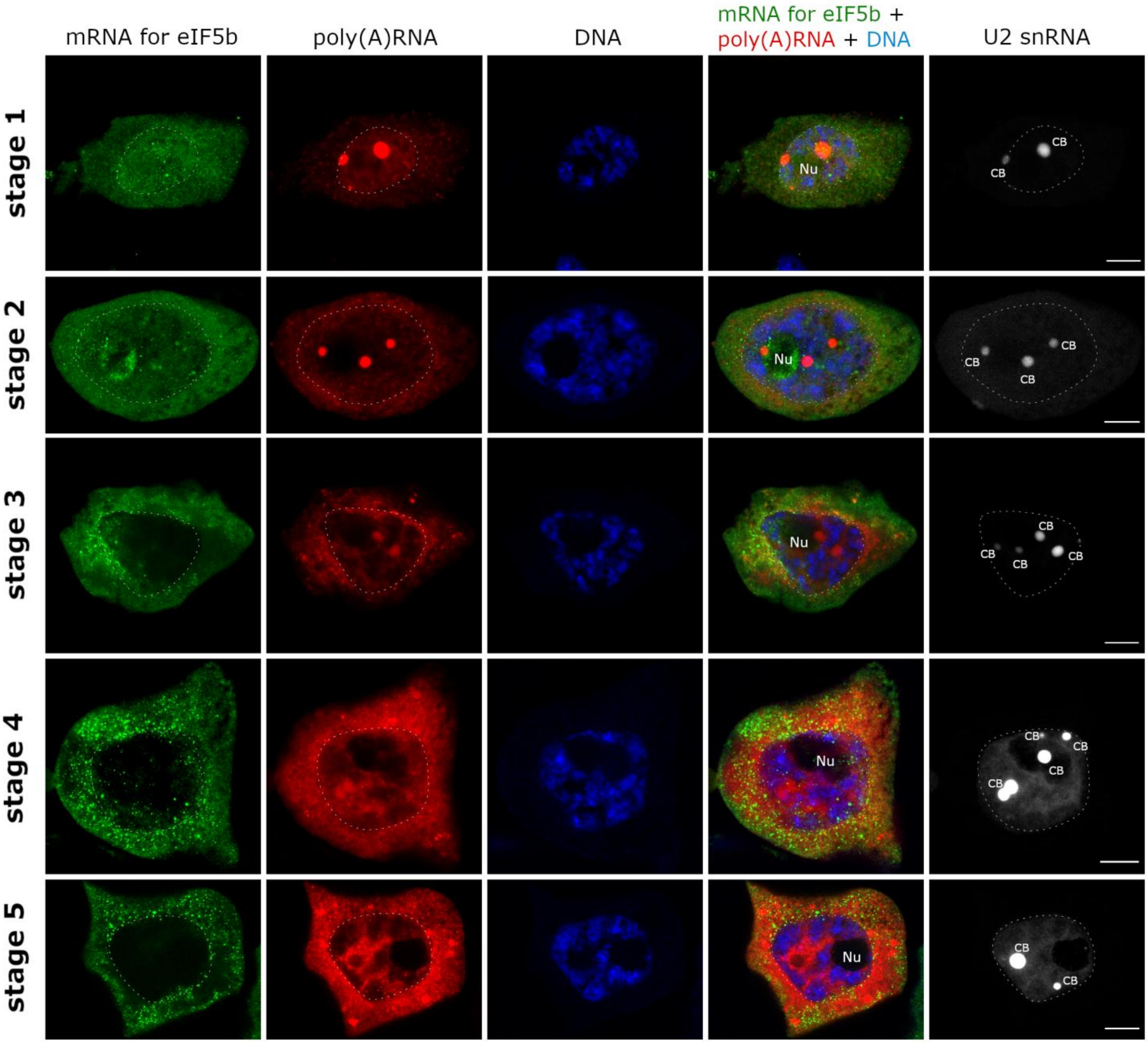

Figure S4. The second pattern of mRNA synthesis and export. Distribution of eIF5b mRNA during poly(A)RNA cycle. eIF5b mRNA (green) is retained in the nucleus until the 2nd stage of poly(A) cycle. The export of eIF5b mRNA starts in the third stage and it is observed in the cytoplasm until the end of the cycle. Bar – 10 µm.

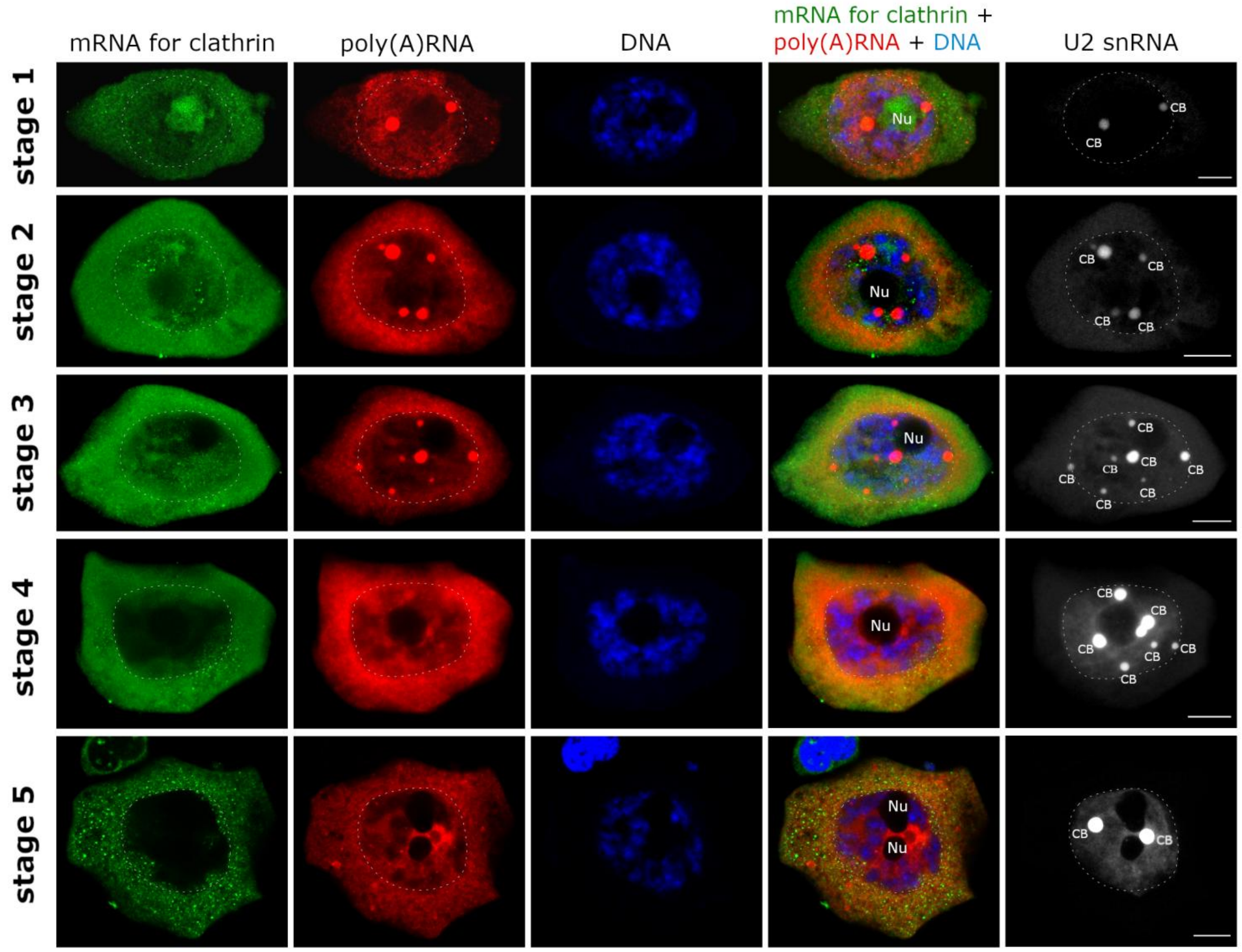

Figure S5. Alternative pattern of mRNA synthesis and export. Distribution of clathrin mRNA during poly(A)RNA cycle. Clathrin mRNA (green) is present in the nucleus up to the 4th stage of poly(A) cycle, while export starts in the second stage. The mRNA is present in the cytoplasm throughout the cycle. Bar – 10 μm.

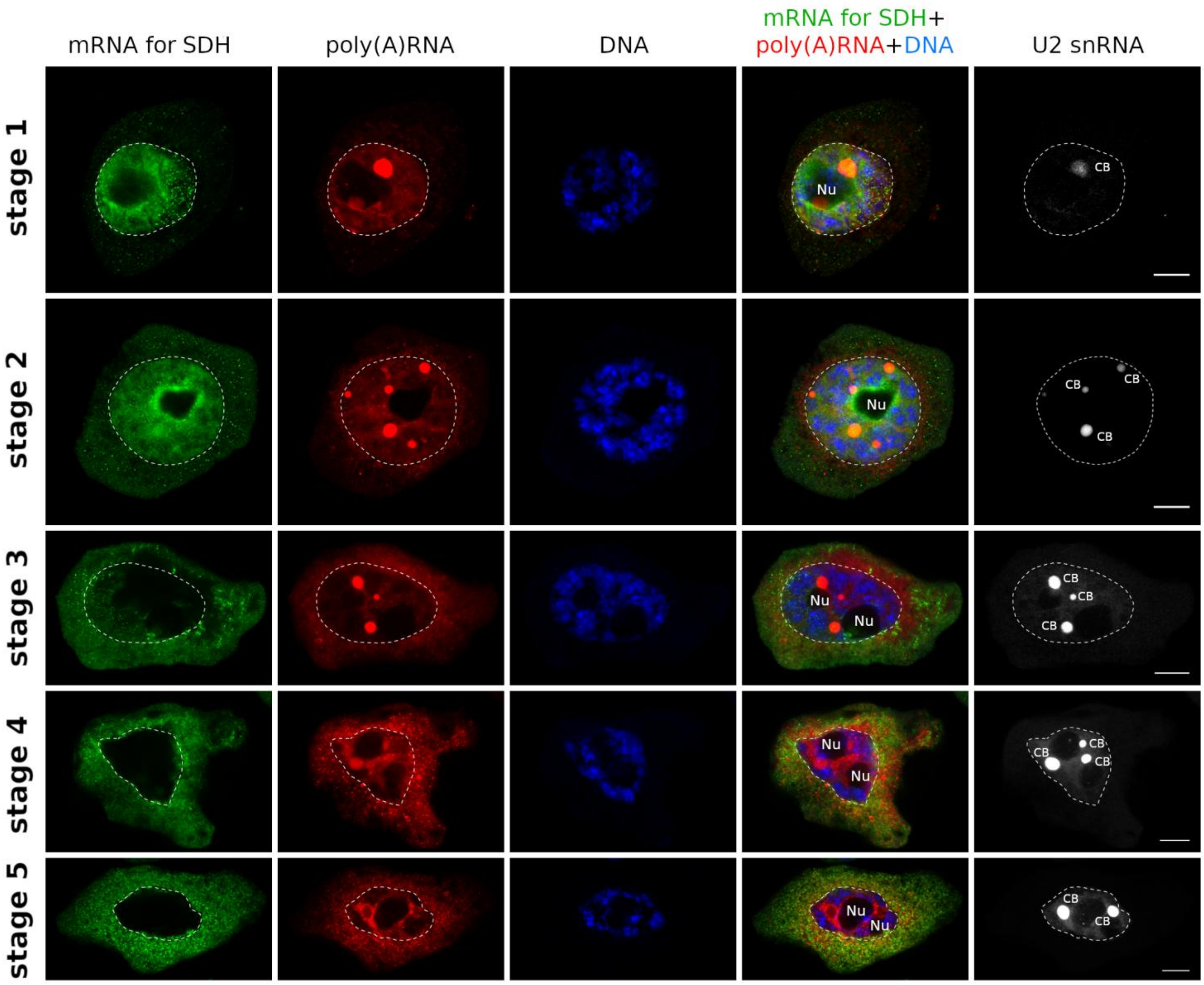

Figure S6. Alternative pattern of mRNA synthesis and export. Distribution of SDH mRNA during poly(A)RNA cycle. SDH mRNA (green) is present in the nucleus up to the 3rd stage of poly(A) cycle, and export starts in the second stage. The mRNA is present in the cytoplasm from the second stage until the end of the cycle. Bar – 10 µm.
